# Understanding the audiological care of patients with co-existing dementia or mild cognitive impairment and hearing loss in the United Kingdom National Health Service: A qualitative study

**DOI:** 10.1371/journal.pone.0327248

**Published:** 2025-06-25

**Authors:** Sian Calvert, Aaron Chitty, Alex Langdon, Emma Broome, Helen Henshaw, Sarah Somerset, Tom Dening, Eithne Heffernan

**Affiliations:** 1 Hearing Sciences, Mental Health and Clinical Neurosciences, School of Medicine, University of Nottingham, Nottingham, United Kingdom; 2 National Institute for Health and Care Research (NIHR) Nottingham Biomedical Research Centre, Nottingham, United Kingdom; 3 Mental Health and Clinical Neurosciences, School of Medicine, Faculty of Medicine and Health Sciences, University of Nottingham, Nottingham, United Kingdom; UFPE: Universidade Federal de Pernambuco, BRAZIL

## Abstract

**Background:**

Hearing loss is common among people living with dementia and can exacerbate the symptoms associated with dementia. The effective management of hearing loss can positively impact quality of life and help alleviate dementia-related symptoms. Little is known about current audiological pathways in the National Health Service (NHS) for adults living with dementia or mild cognitive impairment.

**Objectives:**

To understand the current NHS audiological care pathways for adults living with dementia or mild cognitive impairment.

**Design:**

Qualitative study

**Results:**

Thirty-three NHS audiologists in the United Kingdom (UK) completed a qualitative survey about current adult audiological care pathways for people living with dementia or mild cognitive impairment, and 14 of those also participated in a follow-up interview. Data from the surveys and interviews were combined and analysed using reflexive thematic analysis. The key findings included the importance of person-centred care, the active involvement of carers, and the need for tailored approaches, including using adapted and additional tests to assess hearing loss while offering a variety of hearing interventions. Audiologists expressed a desire for more service integration, such as domiciliary visits, and emphasised the importance of adapting environments and practices, such as dementia-friendly spaces and routine dementia education for staff, to better support individuals living with these co-existing conditions,

**Conclusions:**

These findings can help inform the development of formal clinical practice guidelines and evidence-based training to support audiological care for people living with dementia and mild cognitive impairment in the United Kingdom.

## Introduction

Approximately 55 million people worldwide live with dementia, including 1 million in the United Kingdom (UK) [1,2]. An estimated 20% of adults aged 65 or above are living with mild cognitive impairment (MCI) [3], a condition characterised by a degree of cognitive decline beyond what is typical of ageing, which can be a sign of a disease that causes dementia [4]. Yet not everyone who is diagnosed with MCI will go on to develop dementia [5]. Many people living with dementia or MCI also live with hearing loss. Hearing loss is a progressive condition that affect 20% of the global population [6]. The number of people living with these long-term conditions is growing with increasing life expectancy.

Dementia and hearing loss can significantly impact quality of life. Hearing loss is ranked as the third leading cause of years lived with disability globally [7], and dementia is reported as a major cause of global disability and dependency among older people [8]. These conditions can lead to communication difficulties, social withdrawal, and poor psychological wellbeing [9–11] additionally, hearing loss can exacerbate the behavioural symptoms associated with dementia [12]. It can be hard to differentiate the signs of hearing loss from those of dementia, meaning one condition may mask the other [13]. For example, someone may not respond to a question due to not hearing the speaker or due to having difficulties processing the question and producing a response. The overlapping symptoms of dementia and hearing loss can impact the diagnosis and management of the conditions. For example, audiological tests like pure-tone audiometry (PTA), which rely on behavioural responses to measure hearing thresholds, can be particularly challenging for people living with dementia (PLWD) due to difficulties in following procedural instructions [14].

The National Health Service (NHS) is a publicly funded healthcare system in the United Kingdom (UK), providing a wide range of medical services free at the point of access for UK residents [15]. It plays a central role in delivering primary care (e.g., dentistry, general practice), secondary care (e.g., audiology, emergency care), specialist services and long-term care. In England, hearing services are primarily delivered through NHS audiology departments and, in some cases, through accredited providers in the community and Ear Nose Throat (ENT) departments. In the UK, the typical NHS hearing healthcare pathway for adult-onset hearing loss (i.e., the structured process patients follow for assessment, treatment, and follow-up) begins with a referral from primary care (or self-referral) to audiology services [16]. Patients then typically undergo an audiological assessment, after which one or two hearing aids may be prescribed if needed. The NHS provides these free at the point of care. A follow-up should then be offered 6–12 weeks after hearing aid fitting [16]*.* The early identification of hearing loss in PLWD or MCI is crucial to ensure they can access appropriate support and hearing interventions promptly. National UK guidelines (National Institute for Health and Care Excellence (NICE), NG98) recommend that PLWD should be referred for a hearing assessment every two years, if they have not already been diagnosed with hearing loss [16]. The guidelines do not provide detailed guidance on how these assessments should be conducted (e.g., which diagnostic tests to use, which frequencies to test), nor how hearing loss should be explicitly managed in the context of co-existing dementia. For all patients diagnosed with hearing loss, the guidelines suggest that they should contact audiology if they notice any further changes in their hearing, as there is no specific guidance on retesting. Providing hearing interventions for adults with cognitive impairment can help improve communication, social engagement, and dementia-related behavioural challenges [17,18]. However, PLWD, MCI or informal carers (e.g., family members) may lack awareness of deteriorating hearing and/or fail to seek help for any difficulty experienced. Thus, PLWD have a high rate of untreated hearing loss [19,20]. Additionally, PLWD or MCI can sometimes find hearing aids difficult to use and maintain [21] and informal carers often play a key role in supporting the consistent and effective use of hearing aids [22].

Little is known about the hearing healthcare pathway for PLWD or MCI, who are assessed and managed in adult NHS adult audiology services. Although there is some new guidance about how to discuss the link between dementia and hearing loss [23], there are no national guidelines in the UK for how to assess and manage hearing loss for the growing demographic of people living with co-existing hearing loss and dementia or MCI [24]. There is a lack of detailed guidance on adapting audiological assessments and interventions and supporting hearing device use for PLWD or MCI. While international best practice recommendations have been published for assessing and rehabilitating hearing loss in PLWD [24], they are broad and do not provide an overview of current practice in the UK NHS, meaning that both knowledge and guidance for the audiological care of this population remains limited. Therefore, this qualitative study aims to explore NHS audiologists’ perspectives on current clinical service provision in their UK audiology services for PLWD or MCI.

## Materials and methods

### Study design

This qualitative study comprised (i) a survey with audiologists to examine current practices for PLWD or MCI in UK NHS adult audiology services and (ii) follow-up interviews to develop an in-depth understanding of these practices. Ethical approval was obtained from the University of Nottingham Faculty of Medicine and Health Sciences Research Ethics Committee (FMHS 438−0122). All participants provided informed electronic consent. This study has been reported in accordance with the Reflexive Thematic Analysis Reporting Guidelines [25].

### Participants and recruitment

Participants were recruited using purposive sampling. Purposive sampling is a non-random sampling technique where participants are selected based on specific characteristics, knowledge, or experiences that are relevant to the research aim or question [26]. Audiologists were sought from at least 15 NHS audiology services with representation from different regions of the UK. Recruitment channels included leaflets at national audiology conferences, emails to UK professional networks, social media posts, digital newsletters (e.g., British Academy of Audiology) and professional websites (e.g., British Society of Audiology). Participants were eligible for inclusion if they were (i) 18 years or older and (ii) an audiologist in an NHS audiology service.

Follow-up interviews were conducted by SC and EH with a subset of survey participants who expressed an interest in participating and were selected through purposeful sampling to ensure diverse perspectives were collected from different geographical areas of the UK and with different areas of expertise. Not all individuals who expressed interest in participating were interviewed; participants were contacted based on their geographical location to ensure representation from diverse service settings, and their areas of expertise were reviewed from the survey responses to help provide a broad spread of relevant knowledge and perspectives. Recruitment was via email and ceased once insights were collected from a range of clinics in different geographical locations. Data collection occurred between 1^st^ October 2022 and 30^th^ June 2023.

### Data collection

#### Stage 1: Qualitative survey.

Participants completed a 15-minute qualitative questionnaire (S1 File) via Online Surveys (www.onlinesurveys.ac.uk) or paper-and-pen. Participants were provided with an Information Sheet, which informed them of the nature and objectives of the study. The survey comprised 35 questions, the majority of which were open-ended. Participants were asked to provide information on demographics, the composition of service, a description of usual care within their audiology service, the assessment and management of PLWD or MCI, and any specialist dementia training undertaken. Ten researchers and audiologists piloted the survey, which led to the removal of overlapping questions and those deemed less essential to the research question, helping to keep the survey a manageable length for participants.

#### Stage 2: Interviews.

The interviews were video recorded online using Microsoft Teams and transcribed verbatim with identifiable information removed. Their mean duration was 40.8 minutes (range 32–60 minutes). The interview topic guide was developed by researchers and audiologists (S2 File) based on the research question and aims of the study, to address a gap in the literature about NHS audiology services for PLWD or MCI. Field notes were also taken during the interviews.

### Analysis

Data from the surveys and interviews were combined, and a record of participant numbers was kept, to track individuals across the survey and interview datasets. Responses were analysed following Braun and Clarke’s established six-stage reflexive thematic analysis procedure led by SC [27]: Familiarisation; Coding; Generating initial themes; Reviewing themes; Defining and naming themes; and Write-up. This process included iterative cycles of coding, theme development, and reflection to identify patterns of shared meaning and illustrated the richness and complexity of the data. The main themes were shaped through a process of ongoing engagement with the data, whereby initial codes were collated, reviewed, and refined into broader conceptual groupings. The analysis was complete when the themes were coherent, well-developed, and provided a meaningful picture of the data.

To strengthen the rigour of our analysis, we engaged in peer debriefing [28], by discussing themes and their definitions, ensuring a comprehensive examination of the data from multiple perspectives. This led to changes in the theme names and the order of presentation of themes to support a coherent narrative of the data. SC, who led the qualitative analysis, is a mixed methods early career researcher and psychologist with experience in thematic analysis. AC, AL EH, EB and SS also contributed to the qualitative analysis who were medical students and experienced qualitative researchers based in hearing sciences. Please see supporting materials for the reflexivity statement (S3 File).

### Patient and public consultation

Nine Patient and Public Involvement (PPI) representatives with lived experience of hearing loss and/or dementia were consulted during two online workshops and two one-to-one consultations. They reflected on the qualitative results and made recommendations for future clinical practice. Such consultations can help ensure that research outcomes align with the real-world experiences and needs of those affected by health conditions, thereby enhancing the relevance and applicability of the findings [29]. Six representatives were living with dementia and/or hearing loss, and five were supporters (e.g., relatives) of someone living with dementia and/or hearing loss (representatives could be both someone living with hearing loss and a supporter). They were aged 35–95 years old and included six women.

## Results

### Participant characteristics

A total of 39 participants completed the survey. Six responses were excluded because they were not UK NHS audiologists. Of the 33 eligible participants, 14 also participated in an interview (Table 1).

**Table 1:** Participant characteristics.

Characteristics	Survey (N = 33)	Interview (N = 14)
Gender		
Male	5	3
Female	28	11
Age Group		
18-29 years old	6	3
30-39 years old	10	4
40-49 years old	7	3
50-59 years old	9	3
60-69 years old	1	1
Region of NHS Adult Audiology Service in the UK		
London	3	3
North West England	4	2
Yorkshire	2	2
East Midlands	2	2
West Midlands	3	0
South East England	3	0
East of England	2	0
South West England	3	2
Scotland	3	2
Wales	2	1
Northern Ireland	1	0
Average years of experience (SD)	16.46 (11.26)	16.43 (10.71)
Area(s) of expertise^*^		
Tinnitus	6	3
Hyperacusis	1	1
Vestibular	3	3
Paediatrics	4	1
Learning disabilities or complex needs	5	2
Dementia	8	6
Adult rehabilitation	3	1
None stated	9	1

* Area(s) of expertise was a free text response (S1 File) and responses were tallied.

### Context

Of the 33 responses, 14 stated that their NHS audiology service offered a special clinical pathway for PLWD or MCI, i.e., the structured process patients follow for assessment, treatment, and follow-up specific to PLWD or MCI. The specialised pathways varied in their approach to how PLWD or MCI were assessed and managed, and access to some specialist pathways was impacted by whether someone had a formal diagnosis of dementia.

### Qualitative findings

Three themes were identified (Table 2), illustrating audiologists’ experiences supporting PLWD or MCI alongside the adaptations they aspire to introduce in future practice. No regional differences in participant responses were identified during the analysis.

**Table 2:** Overview of themes.

Theme	Description	Example quotes
Overarching principles for providing audiological care	This theme outlines key principles and considerations for delivering audiological care to PLWD or MCI, highlighting the importance of person-centred care, active involvement of carers, and the variability in dementia training undertaken by audiologists.	*“Ideally…both the patient and their carers… know how to do all the management, put the hearing aids in, take them out, keep them clean, change the batteries, all those things and just make sure that they know how to contact us if they need to”* (Female Audiologist, aged 30-39, based in the East Midlands, Interview).
Adapting approaches to provide audiological care	This theme highlights the adaptations audiologists make to provide tailored care to PLWD or MCI, such as modifying hearing assessments and offering a diverse range of hearing interventions.	*“More variety of tests that could be used if required”* (Female Audiologist, aged 30-39, based in the South West of England, interview). *“Use of ALDs [assistive listening devices] instead of aids when needed.”* (Female Audiologist, aged 40-49, based in Scotland, Interview).
Re-imagining audiological care	This theme explores ways audiologists would like to change how they provide hearing care to PLWD or MCI. Proposed improvements included offering domiciliary visits and having specialist dementia-friendly spaces.	*“Do more domiciliary visits...just checking up their environments and, going to check on them in, in their place of where they live”* (Female Audiologist, aged 30-39, based in Yorkshire, Interview). *“But it’s basically adjusting the environment [in audiology] to like specifically for people with dementia”* (Female Audiologist, aged 30-39, based in the East Midlands, Interview)

### Theme 1: Overarching principles for providing audiological care

#### Person-centred approach.

Participants consistently reported the importance of person-centred care. It was agreed that hearing loss management should be tailored to suit the individual needs and lifestyles of PLWD or MCI:

*“The patient’s morning routine is thought about and discussed, and how the hearing aid use (putting in, taking out, changing batteries, or charging the aids) can fit in with this. Reminders can be set up on the patient’s or carer’s phone or iPad.”* (Female Audiologist, aged 18-29, based in Yorkshire, Survey)

One of the challenges audiologists communicated to delivering care was the high number of PLWD or MCI who failed to attend scheduled appointments. Participants suggested that appointments could be adapted by scheduling them at a time that fits with the patient’s lifestyle and adjusting the length of appointments, which could be either having longer appointments or several shorter appointments to meet the patient’s needs.

*“Standard assessment and [hearing aid] fit is 90 minutes, and we allowed two hours for a dementia test and fit because it gives you that time to go through things much slower and in much more detail and repeat things so that they’ve understood it.”* (Female Audiologist, aged 18-29, based in the South West of England, Interview)

Some audiologists reported personalising care by taking a multidisciplinary approach by having “*joint appointments with specialist [SLT] team or other healthcare professionals”* (Female Audiologist, aged 50–59, based in London, survey) to provide comprehensive and coordinated care.

#### Dementia training.

Most participants reported that some dementia training had been provided within their service; however, the training across clinics differed. Training was often offered internally by the hospital trust and/or colleagues:

*“They have, [name of area], has developed a whole dementia skills training programme [for NHS staff]. It used to be done face-to-face with big booklets, and now it’s mainly online.”* (Female Audiologist, aged 60-69, based in Scotland, Interview)

Some departments also had a dementia specialist audiologist *“The dementia specialist [audiologist] has given broad training about dementia, audiology adaptations, tips, and tricks.”* (Female Audiologist, aged 18-29, based in Yorkshire, Survey)

Shadowing was also mentioned as a method for staff to gain practical experience in working with PLWD:

*“When someone joins the dementia team, they’ll have a certain number of sessions [where] they come into the same appointments and…watch or do it together with that person just to get a bit more training and experience of that sort of patient group and to see the kind of adjustments that you might make to the appointment.”* (Female Audiologist, aged 30-39, based in the East Midlands, Interview)

High staff turnover and the use of bank (temporary) staff were described as a challenge to ensure that all have appropriate dementia training, and it is often unknown whether the training delivered was accurate or appropriate:

*“The staff numbers and the staffing… changes so often…everybody will probably be new because rotations are so frequent, staff move in and out so quickly and there’s lots of bank staff. It’s very difficult to know that everybody is [dementia] aware.”* (Female Audiologist, aged 30-39, based in Yorkshire, Interview)

“*All front-facing staff should be at least dementia aware, but the problem is no one actually like checks whether these recommendations are ever implemented”* (Female Audiologist, aged 60-69, based in Scotland, Interview).

#### Carer involvement.

Participants underscored the importance of having a carer (formal or informal) present when conducting an appointment with PLWD or MCI. They felt that carers could help to corroborate whether the patient is wearing and maintaining their hearing aids and can help ensure that PLWD or MCI receive adequate support to manage their hearing loss at home.

*“Involving the carers and making sure that they know as much as a patient*” (Female Audiologist, aged 18-29, based in the East Midlands, Interview)

However, some participants cautioned that a balance must be struck to ensure that information is provided to the carer without excluding or undermining the PLWD or MCI. Participants felt that appointments could be challenging when someone did not attend with a carer, or the carer may also be living with dementia:

*“Quite shocking to be honest, is the number of people who are living with dementia by themselves with absolutely no family or care support at all, or couples who both had dementia looking after each other”* (Female Audiologist, aged 60-69, based in Scotland, Interview)

Participants described how patients residing in care homes relied on the knowledge and skills of paid carers to manage their hearing loss. Some felt that care staff have limited knowledge and skills regarding hearing loss management, which was also impacted by the high staff turnover in this sector:

*“The one thing that worries me is care homes: staff training and staff awareness…it feels like just this constant instruction and constant trying to share information with staff and then it’s different staff [in each appointment]”* (Female Audiologist, aged 40-49, based in the North West of England, Interview)

### Theme 2: Adapting approaches to providing audiological care

#### Hearing assessment.

Participants described how PLWD or MCI often required additional or different tests that were not routinely used in usual care. There was a lack of guidance for audiologists about what hearing assessment would be best for PLWD or MCI. Audiologists commented on how they tailored audiological testing. For example, many adapted pure tone audiometry (PTA), such as by testing fewer frequencies to reduce fatigue or asking for verbal responses to tones, which can be more suitable than button press responses for some PLWD or MCI:

*“Often do verbal response PTA, with patient-facing me to keep them engaged. Modify PTA [and] response method as required.”* (Female Audiologist, aged 30-39, based in the East Midlands, survey)

Some reported that instead of standard assessments, they may opt to use objective measures (e.g., otoacoustic emissions, visual reinforcement audiometry) as there can be challenges related to PLWD understanding verbal instructions given within hearing assessments or not fully articulating their hearing difficulties. Some audiologists reported that patients with MCI/dementia were: “*seen by a senior member of the team with flexibility to adapt test method[s]*” (Female Audiologist, aged 30–39, based in the East of England, Survey). Several audiologists noted that when there is not a dementia specialist audiologist, the paediatric team is involved in assessing patients with dementia due to their specialist skillset:

*“[In] paediatrics, they tend to be doing much more complex stuff naturally… the kind of differential diagnosis and the investigative side of things tend to be a bit more involved and tend to require a bit more thought and…that combined with the counselling is what makes [paediatric] staff sometimes a natural fit.”* (Male Audiologist, aged 30-39, based in the East of England, Interview)

Despite the insights provided by audiologists on how audiological assessments may differ for PLWD, it remained unclear whether these methods were successful in supporting hearing assessments for this population:

*“…there’s no research out there to evidence why we’re doing things and why we might not do things.”* (Female Audiologist, aged 30-39, based in Yorkshire, Interview)

It was also reported that some patients do not present for an audiological assessment until they are living with more advanced dementia. The timeline of an audiology referral was suggested to be related to the perceived lack of knowledge of hearing loss among primary care professionals. For example, a participant (Female Audiologist, aged 60–69, based in Scotland Interview) said:

*“A* *lot of, particularly in other medical fields,…doctors and nurses, their knowledge of hearing loss, if they have any, is about 20 years out of date.”*

This was perceived to impact the diagnosis and subsequent management of hearing loss. One participant stated: “*We should be seeing people right the start of their [hearing loss] journey”* (Female Audiologist, aged 60–69, based in Scotland, Interview) as audiologist believe that PLWD would have better outcomes, for example, increased hearing aid use, the earlier they are assessed in audiology.

#### Hearing intervention.

Participants reported that the most common management option for people living with co-existing dementia and hearing loss is the prescription of hearing aid(s). However, an exception was one audiologist who commented that they would “*Usually only offer hearing aids to adults without cognitive impairment”* (Male Audiologist, aged 40–49, based in the South West of England, Survey). Audiologists discussed adaptions that could be made to support the use of hearing aids in PLWD or MCI, for example, *“low battery indicator lights and tamper resistant battery drawers [on hearing aids]”* (Female Audiologist, aged 30–39, based in Yorkshire, Survey). These adaptations are designed to address common challenges, such as carers not always knowing when hearing aids need recharging or, batteries need replacing, or batteries being accidentally removed when the compartment is opened. Additionally, many clinics offered free replacement hearing aid(s) for PLWD who lose them, as this can be common:

*“There’s no admin charge for anyone who’s got dementia, whereas there might be an admin charge applicable to…[the] general population.*” (Female Audiologist, aged 30-39, based in the East Midlands, Interview)

When reviewing or updating prescribed hearing aids, participants noted that it was important to minimise changes, for example, using the same style of hearing aid.

*“I try not to change too many things compared to what they had before, unless they of course, asked me to do so…avoid changes, if possible, [as] it is quite traumatising.”* (Female Audiologist, aged 40-49, based in Scotland, Interview)

The type of hearing intervention audiologists provide to PLWD may depend on the stage of dementia. One participant stated, “*hearing aids are not always the answer*” as PLWD *“just don’t want it”* (Female Audiologist, aged 18–29, based in the South West of England, Interview) or find hearing aids challenging to manage. Some participants described how in patients with late-stage dementia, they instead prioritise “*making sure that patients [are] safe* (Female Audiologist, aged 50–59, based in London, Interview) by offering other types of intervention, which may include assisted listening devices.

#### Ongoing care.

Audiologists discussed making adaptions to ongoing and follow-up care. This includes providing planned reviews for PLWD due to the changing circumstances associated with the condition:

*“After initial reviews, I put them on a yearly review waiting list because for people with dementia…things often change quite quickly… you may have fitted the hearing aids in January, and by February, they have fallen over, they’ve gone into hospital, they’ve lost their hearing aids, [or] they’ve been transferred to a care home.”* (Female Audiologist, aged 60-69, based in Scotland, Interview)

*“Most [other patients] have open reviews [i.e., they are required to book their own]. More severe dementia [patients] would be offered planned reviews.”* (Female Audiologist, aged 50-59, based in the West Midlands, Survey)

Several audiologists adjusted the timeline of ongoing care. For example, one noted that reducing the hearing aid follow-up period, from 12 to six weeks, ensured any issues were addressed more promptly, which helped to support long-term hearing aid use:

*“We are doing the six-week telephone review with these with these patients, whereas before we would just do a 12-week telephone review…unless there [were] problems.”* (Female Audiologist, aged 40-49, based in Wales, Interview)

Audiologists also described signposting patients to local charities that provide additional support, although these appeared to be dementia-related rather than hearing-related:

*“We’re also really lucky that we’ve got a charity…which is specifically for families and adults with dementia…where sort of you can signpost to support.”* (Female Audiologist, aged 40-49, based in the North West of England, Interview)

## Theme 3: Re-imagining audiological care

### Enhancing access and collaboration.

Audiologists expressed a desire for increased time within their schedules to provide domiciliary appointments and to support patients residing in care homes who may not otherwise receive audiology care. This would also provide an opportunity to raise awareness about hearing loss and educate care home staff, thus improving hearing care in these settings:

*“Do more domiciliary visits and do more nursing home visits for education and to show the impact of hearing loss and show the benefits of having hearing aids.”* (Male Audiologist, aged 50-59, based in London, Interview)

Furthermore, a participant (Female Audiologist, aged 30–39, based in the South West of England, Interview) stated they would “*like a mobile dementia friendly clinic where you’re going out to them [in the community]”* to bring audiology services directly to patients in the community, ensuring they are accessible to those who cannot easily travel to clinics.

Audiologists also discussed the benefits of collaborating with memory services, as this is not routinely done. “*We wanted to have some sort of joint collaboration* (Female Audiologist, aged 50-59, based in London Interview) to ensure hearing loss is not missed as “*the older you are the more like you are to have a hearing loss kind of thing it’s just encouraging really the memory assessment staff really to push it [to have a hearing assessment]”* (Female Audiologist, aged 50–59, based in London, Interview). Encouraging memory assessment staff to recommend hearing assessments could help integrate audiology services more seamlessly into dementia care pathways, ensuring patients receive holistic support.

### Improving dementia-friendly audiology practices.

A common view was the need for routine education about working with PLWD for staff within audiology services and assessment of implementation of education:

*“It would be really nice for everybody in audiology to be…dementia aware, so to get that dementia training, so that people, especially…new graduates, don’t freak out if they see someone’s got dementia [who doesn’t] act in the way that they want or they can’t get hearing test out.”* (Female Audiologist, aged 30-39, based in Yorkshire, Interview)

Another recommendation was the development of dementia-friendly rooms, as these spaces were not common within audiology clinics and “*aren’t necessarily the most comfortable or familiar environments…that can sometimes be a barrier to effectively testing*.” (Female Audiologist, aged 30–39, based in Yorkshire, Interview). Creating spaces tailored to the needs of PLWD was identified as a way to enhance both the quality of care and patient experience.

Many commented on the need for technological innovation, particularly the development of hearing aids that could address some of the challenges audiologists have encountered with PLWD or MCI. For example, reducing water damage to hearing aids by *“having hearing [aids] that could go through the wash”* or reducing the number of lost aids by having *“GPS in hearing aids so if they get lost, they can be found easily”* (Female Audiologist, aged 40–49, based in the North West of England, Interview).

## PPI representatives’ reflections

PPI representatives reflected on the findings and made recommendations for future practice. They agreed that tailored audiology care for PLWD or MCI is crucial, and all services should have a specialist dementia pathway. They proposed that appointments should be shorter, more frequent, and face-to-face. Appointments should occur in an accessible and calm setting within the audiology service or the person’s residence. Being seen by a paediatric audiologist is appropriate, as they possess the necessary skill set, as long as the audiologist engages with the PLWD in a respectful manner. There should be flexibility in scheduling appointments, such as ensuring those who miss appointments are offered a new one as soon as possible. Offering hearing aids to all eligible patients, including those PLWD or MCI, was strongly advocated, especially to prevent social withdrawal. Dementia awareness training for audiologists should be more in-depth, and this should be expanded to all patient-facing staff, including receptionists. Audiologists require greater collaboration with other services (e.g., general practice, care homes, memory clinics) and should train staff in those services about hearing loss. Old-age psychiatrists and GPs could play a key role in supporting PLWD or MCI in obtaining hearing aids, as patients tend to be familiar with them and are likely to follow their recommendations. Involving carers in appointments and educating them about hearing loss is vital. However, audiologists must not overlook PLWD or MCI when carers are present. Audiologists must develop approaches for those without carers or those who do not view their relative/spouse as their carer. Finally, audiology services should use outcome measures to assess the effectiveness of adaptations for PLWD or MCI. S4 File provides a visual representation of the PPI reflections and recommendations for future audiological care of PLWD or MCI

## Discussion

To our knowledge, this is the first study to explore NHS audiologist’s perspectives on current clinical service provision in audiology clinics for PLWD or MCI in the UK. The findings indicate that not all services have a specialist dementia pathway. There was variation in care approaches and adaptations in services with such pathways. These inconsistencies are likely due to a lack of formal guidance on how to effectively provide hearing care for this population.

In audiology services with dementia pathways, care decisions were primarily guided by the audiologist’s professional experience and expertise about what worked best for this population. In the UK, the National Institute for Clinical Excellence guidelines [16] for the care of those with suspected or diagnosed dementia is the primary source of guidance available to audiology services. Formal guidance on detecting, diagnosing and managing hearing loss in PLWD remains an unmet need. Littlejohn et al. [24] developed a set of practice recommendations via consensus with professional stakeholders to manage sensory impairment in PLWD; however, these preliminary guidelines are broad and do not provide an overview of current UK NHS practice.

Our findings show that clinical shadowing and internal training are the primary sources of dementia-related education for staff working within the NHS audiology services included in this research, suggesting variability both in content and approach. In addition, it was unclear how effectively the learning from shadowing or training was incorporated into audiological care. A lack of evidence-based training may lead to unintentional learning of undesirable practices or behaviours that could impact patients’ quality of care [30]. Nevertheless, there are some pockets of excellence, with some audiologists who specialise in complex cases, including dementia, seeking active development through networks set up to facilitate learning and development, such as the British Society of Audiology (BSA) ‘Dementia Network’, which is an educational and peer support network coordinated and led by the BSA Cognition in Hearing Special Interest Group. Findings from this study suggest that training to increase specialist skills and clinical expertise for audiologists working with PLWD would be of benefit. Indeed, the importance of dementia training and awareness across all audiology services has been highlighted in a review of hearing assessment and rehabilitation for PLWD [31]. Research has shown that dementia training can improve staff knowledge and confidence, leading to better outcomes for PLWD [32]. However, it is important that training is evidence-based and efficacious, considering the heterogeneous nature of the workforce [33]. Additionally, if all audiologists receive dementia-specific training, it could ensure all services can provide appropriate hearing care to PLWD or MCI, helping to mitigate the postcode lottery effects on access to care.

Living with both dementia and hearing loss presents complex challenges that necessitate flexible clinical pathways to ensure the delivery of optimal hearing healthcare. Audiologists emphasise the importance of providing person-centred hearing care throughout the pathway, tailoring both the assessment, management and ongoing care to meet the unique needs of PLWD. For example, PLWD may struggle with articulating their hearing difficulties or find following the verbal instructions of a hearing test challenging. To address these challenges, PLWD should be supported by audiologists with specialised skills, such as those trained in dementia care or paediatric audiologists. These clinicians offer flexible and adaptable approaches that could meet the needs of PLWD.

The findings indicate that while hearing aids are the primary intervention for hearing loss, and that hearing aids should always be offered where appropriate, alternative options should be considered when hearing aids are *not* suitable for an individual. A broader exploration of alternative hearing interventions is essential to ensure that management strategies align with each person’s individual circumstances and preferences. The present study identified varying approaches to ongoing hearing care for PLWD, with formal and informal carers playing an important role in managing hearing loss outside clinical care. There is no specific guidance for follow-up care for this PLWD or MCI, although recommendations suggest that more frequent reviews would be pragmatic for those with impaired cognition [31]. In the UK, the NICE guidelines [16] suggest that PLWD should have their hearing assessed every two years if not previously diagnosed with hearing loss. However, there can be discrepancies between guidelines and practice [31]. As reported in this study, often, the onus is on the patients themselves to seek help if facing hearing challenges.

The findings also indicated that patients may benefit from increased collaboration between audiology and other health services (e.g., primary care, memory clinics) and that staff in other services should be aware of hearing loss and how it can impact PLWD. Research shows that untreated hearing loss can impact the symptoms of dementia and increase the challenges of managing dementia [13,34], making coordinated care essential. Staff in these services should be trained to recognise hearing loss, as evidence indicates that increased awareness can lead to earlier intervention and improved quality of life for PLWD [35].

Audiologists imagined, from their perspective, what NHS hearing health care could look like in the future to better support PLWD. There must be consistency in dementia pathways in audiology to ensure equitable, reliable, and effective care is offered to all patients living with dementia. However, further research is warranted to understand what needs to be part of the pathway to best support positive patient outcomes. Audiologists articulated that the environment where care is offered should be dementia friendly to support positive patient experience, and there should be flexibility in appointments; for example, considering how to support patients to attend appointments (e.g., appointments at a time that best suits them) and being able to offer appointments in care homes. Many people who reside in care homes are living with dementia and hearing loss [36] and are an underserved population within audiology services, meaning they may not receive appropriate hearing care. Previous research has highlighted that hearing rehabilitation, including screening and referrals, is uncommon in care homes [37].

### Strengths and limitations

This study contributes to the limited evidence base of how hearing loss in PLWD or MCI is assessed and managed in NHS audiology services. To enhance the credibility and validity of the findings, the authors engaged in peer debriefing during analysis. This approach is used to enhance the rigour, nuance and balance of the interpretation of the data [38,39]. Although we explored the perspectives of audiologists regarding current care in NHS audiology, it is unclear what care private UK audiological providers offer and how this could differ. Future research could investigate these differences and explore the management of other hearing conditions, such as tinnitus, and the care pathways for cochlear implants among PLWD. Furthermore, this qualitative study focused on exploring the perspectives and experiences of a purposefully selected group of NHS audiologists, rather than aiming to represent all NHS audiologists, future research could address this.

### Clinical implications

These findings have several clinical implications for audiology services. Firstly, they demonstrated inconsistency in UK NHS audiology pathways for PLWD or MCI. Even though some of the adaptations currently made to support hearing assessment and management seem appropriate, there is a need for clear guidance to implement best practices alongside measurement of the impact on patient outcomes. Furthermore, variability in dementia education undertaken by audiologists emphasised the requirement for consistent, comprehensive, monitored, dementia-specific training to ensure appropriate care is delivered. It is vital that a person-centred approach is adopted, including the adaptation of appointment times and formats to suit the lifestyle and cognitive abilities of PLWD, with care delivered in dementia-friendly environments. It was considered important to involve carers where possible, whilst respecting the PLWD, and to develop approaches for individuals attending the clinic without carers. Community-based support systems would appear necessary to address the evolving needs of PLWD by providing ongoing, accessible care. Finally, the findings of this study highlight the importance of strengthening collaboration between audiology and other healthcare sectors, such as GPs, memory clinics, and care homes; this could facilitate a more integrated and holistic approach to care. Hearing care may be overlooked in other healthcare pathways without effective collaboration, leading to fragmented care and poorer outcomes for PLWD or MCI. Greater emphasis is also needed on raising awareness of the importance of hearing care amongst all health and social professionals, particularly since hearing is often overlooked despite its critical role in cognitive assessments and healthcare interactions. S5 File provides a visual representation of the clinical implications for hearing care of PLWD or MCI across the patient pathway.

## Conclusions

This study addresses a key gap in the literature by exploring current pathways in audiology for PLWD or MCI. The findings from this study provide valuable insights to help inform the development of formal guidelines and evidence-based tailored training programmes aimed at enhancing audiological care for PLWD and MCI in the UK NHS and could help shape practice guidance for UK professional audiology organisations. Establishing consistent, inclusive pathways and equipping healthcare professionals with the necessary skills and knowledge will support delivering effective, person-centred care. By addressing these needs, audiology services can play a crucial role in improving this population’s quality of life and overall health outcomes.

## Supporting information

S1 File Qualitative questionnaire. (DOCX)

S2 File Interview schedule. (DOCX)

S3 File Reflexivity statement. (DOCX)

S4 File Visual representation of the PPI reflections and recommendations for future audiological care of PLWD or MCI. (DOCX)

S5 File Visual representation of the clinical implications for audiological care of PLWD or MCI across the patient pathway. (DOCX)
