# Supplementary material for: Understanding the audiological care of patients with co-existing dementia or mild cognitive impairment and hearing loss in the United Kingdom National Health Service: A qualitative study
Source: PLoS One. 2025 Jun 25;20(6):e0327248. doi: 10.1371/journal.pone.0327248 (PMC12193678; doi:10.1371/journal.pone.0327248)
Supplement: S1 File — (DOCX) [file pone.0327248.s001.docx]

# Supporting File 1

Qualitative Questionnaire

**Demographics**

What is your profession?

How many years professional experience do you have?

Do you have any particular areas of expertise?

How would you describe your gender?

- Male
- Female
- I would describe it in another way
- Prefer not to say

What is your age group?

- 18-29 years
- 30-39 years
- 40-49 years
- 50-59 years
- 60-69 years
- 70 years and above

Please tell us the name of your audiology service/organisation.

**Adult Audiology Service Composition**

In terms of days/hours of operation, when is your adult audiology service normally open?

Approximately how many direct referrals from primary care does this service receive per month?

- <50
- 51-100
- 101-150
- 150-200
- Other (Please explain)

What is the catchment area (e.g., postcode) for referrals to this service?

Please give us a brief description of the usual care pathway in your service (e.g., activities and timelines related to pre-assessment, assessment, treatment, and follow-up).

**Management of Mild Cognitive Impairment/Dementia – Specialist Pathway**

Does your audiology service offer a specialist clinical pathway for adults with suspected or diagnosed cognitive impairment or dementia?

- Yes
- No
- Unsure

***(If yes above)*** Approximately how many referrals are there to this specialist pathway per month?

***(If yes above)*** Please briefly describe any eligibility criteria that are used to decide if someone should be referred to this pathway (e.g., cognitive test score, case history)?

***(If yes above)*** Who is involved in making referral decisions for this pathway (e.g., dementia specialist audiologist, GP)?

**Management of Mild Cognitive Impairment/Dementia**

*Please write 'not applicable' or 'unsure' if needed.*

Approximately how many staff are involved in providing specialist dementia care in your service?

Do any staff in your service have specialist training in dementia?

- Yes
- No

If yes, please briefly explain (e.g., What type of training? Who provides it?)

Does your service follow a particular guideline or documented procedure for managing patients with cognitive impairment/dementia?

- Yes
- No

If yes, please briefly explain.

Does the length of appointments typically differ between patients with and without cognitive impairment/dementia?

- Yes
- No

If yes, please briefly explain.

What audiological assessments are undertaken for adults with cognitive impairment/dementia in your service?

How do they differ from usual care?

Are cognitive assessments (e.g., MoCA) undertaken in your service?

- Yes
- No

If yes, please briefly explain.

What interventions, treatments or management options do you offer to adults with cognitive impairment/dementia?

- How do they differ from usual care?

What follow-up and ongoing care do you routinely offer to adults with cognitive impairment/dementia?

- How does this differ from usual care?

Are there any other key differences between usual care and care for adults with cognitive impairment/dementia in your service (e.g. differences in history taking, audiologic counselling, outcomes measured, carer involvement)? Please explain below.

Do you ever refer adults with suspected or diagnosed cognitive impairment/dementia to other services?

- Yes
- No

If yes, please briefly explain (e.g. How is this decided? Where are they referred?)

Do you ever provide care in nursing homes/care homes for adults with cognitive impairment/dementia?

- Yes
- No

If yes, please briefly explain.

To be entered into the prize draw for a £50 voucher for completing this survey, please provide your email and/or phone number:

**Thank you for completing the survey**
