# Supplementary material for: Understanding the audiological care of patients with co-existing dementia or mild cognitive impairment and hearing loss in the United Kingdom National Health Service: A qualitative study
Source: PLoS One. 2025 Jun 25;20(6):e0327248. doi: 10.1371/journal.pone.0327248 (PMC12193678; doi:10.1371/journal.pone.0327248)
Supplement: S2 File — (DOCX) [file pone.0327248.s002.docx]

# Supporting File 2

Interview topic guide

1. Please could you tell me about any experience you have of working with patients living with dementia or any particular interest you have in this area?
2. **Pathway:** Please could you give me a brief overview of the service or pathway for patients who are living with dementia within your audiology service.

OR

1. **No Pathway**: Please could you give me a brief overview of what typically happens when patients living with dementia attend your audiology service.
2. Please tell me from your perspective what works well in terms of the management of patients with dementia in your audiology service? And why does it work well?

Possible areas to probe: Consent, hearing assessment, treatment, follow-up care, communication/information given, training, staffing.

- - *What is it about XX that works well?*
  - *Do you think the pathway supports everyone with mild cognitive impairment or dementia? And why?*

1. Please tell me from your perspective are there any areas that need to be improved in terms of the management of patients with dementia in your audiology service.

Possible areas to probe: Consent, hearing assessment, treatment, follow-up care, communication/information given, training, staffing.

- - *What is it about XX that does not work well?*
  - *Is there something else that could be done instead of XX?*

1. If time and resources were unlimited, or there were no restrictions on what you could do for patients, what additional support do you feel would be beneficial for those patients who live with dementia?

*Possible areas to probe:*

- *Training*
- *Standardised guidelines / best practice recommendations*
- *Additional resources*
- *Links with other services e.g. memory clinic*
- *Memory assessment in audiology or hearing assessment in memory clinics*
- *Home visits/visits to care homes*
  - *How do you think this support could best be provided?*
  - *At what point in the pathway could this be provided? E.g., pre-clinical, assess, fit, follow up.*

1. Is there anything you would like to tell us about that we haven’t already discussed?

Additional questions if there is time

1. From your perspective, what are the main barriers and facilitators to optimal audiology care for people living with dementia?
2. Are there any factors that affect decisions within the pathway or about the management of this patient group? e.g., stage of dementia, type of dementia, and carer support.

**Questions for participants that do not work directly with adults**

1. Please tell me briefly about your current job role.
2. Could you tell me about any experience you have working with adults in audiology?
   1. *Does the clinic you work in support both children and adults?*
   2. *Are you aware of any clinical audiology pathway for the management of patients who live with coexisting mild cognitive impairment or dementia within your service?*
3. We are interested to understand what adjustments are made when working with children e.g. testing, and consent.
   1. *Is there anything that we could learn that could be important when considering working with people living with dementia*
4. Is there anything you would like to tell us about that we haven’t already discussed?
