# Supplementary material for: Understanding the audiological care of patients with co-existing dementia or mild cognitive impairment and hearing loss in the United Kingdom National Health Service: A qualitative study
Source: PLoS One. 2025 Jun 25;20(6):e0327248. doi: 10.1371/journal.pone.0327248 (PMC12193678; doi:10.1371/journal.pone.0327248)
Supplement: S3 File — (DOCX) [file pone.0327248.s003.docx]

## Supporting File 3

Reflexivity statement

As researchers, we acknowledge that our professional and personal experiences may influence how we approach and interpret the data. This study was conducted by a diverse team with expertise in dementia and hearing research, qualitative methodologies, engagement with PPI representatives, and personal experience of acquired hearing loss. We recognise that our collective knowledge and experiences could have shaped both the data collection process and its analysis. To address this, regular meetings were held during the interview phase to reflect on the process and refine our approach, supported by the use of reflective diaries. Additionally, throughout the analysis, we engaged in peer debriefing sessions to critically review and discuss the interpretation of findings. This collaborative and reflexive approach enabled us to draw on our varied expertise while remaining vigilant about potential biases, ultimately contributing to a more nuanced and balanced understanding of the study's findings.
