# Supplementary material for: Understanding the audiological care of patients with co-existing dementia or mild cognitive impairment and hearing loss in the United Kingdom National Health Service: A qualitative study
Source: PLoS One. 2025 Jun 25;20(6):e0327248. doi: 10.1371/journal.pone.0327248 (PMC12193678; doi:10.1371/journal.pone.0327248)
Supplement: S4 File — (DOCX) [file pone.0327248.s004.docx]

## Supporting File 4


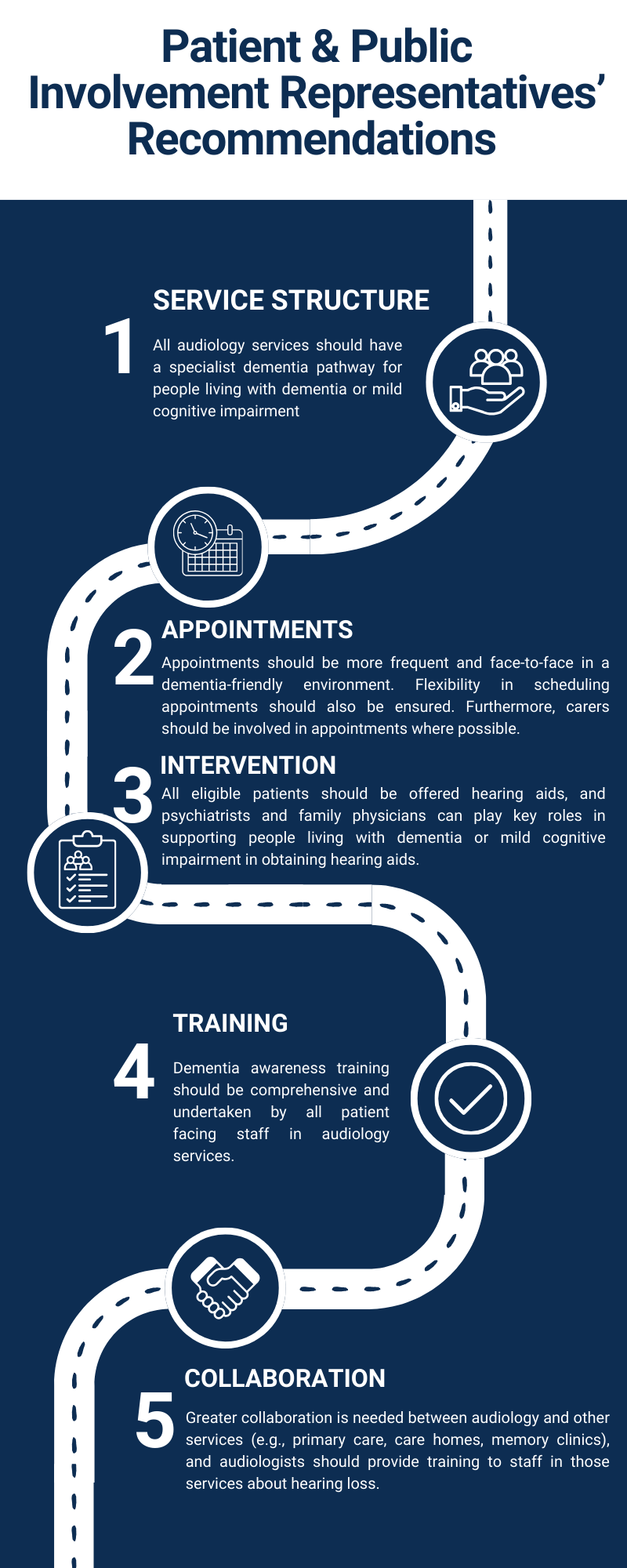


Patient and public involvement representatives’ reflections and recommendations about audiological care of people living with dementia or mild cognitive impairment
